# Family Attitudes towards Multilingualism in Bilingual Education Programs and Their Relationship with Academic Performance

**DOI:** 10.3390/bs14010006

**Published:** 2023-12-21

**Authors:** Jorge González Alonso, Jon Andoni Duñabeitia

**Affiliations:** 1 Nebrija Research Center in Cognition (CINC), Nebrija University, 28043 Madrid, Spain; jdunabeitia@nebrija.es; 2 Center for Language, Brain and Learning (C-LaBL), UiT The Arctic University of Norway, 9019 Tromsø, Norway

**Keywords:** multilingualism, bilingual education, family attitudes

## Abstract

This article reports on a large-scale study investigating the overall perception of multilingualism in the family environment of children enrolled in an English immersion program in primary schools across Spain and the potential relationship between these attitudes and the student’s academic performance. One thousand and one families participated in the study, based on a tailored questionnaire that evaluated three main aspects: (1) parents’ and guardians’ ratings of their children’s language skills; (2) language practices in the home, especially with respect to time allocated to different languages and multilingual practices such as code-switching; and (3) attitudes and general perception of the benefits and drawbacks of multilingualism in socio-economic, cultural, cognitive and professional terms. The complex results from this questionnaire, in addition to providing a more accurate picture of the family environment of students enrolled in these programs, show some significant correlations with academic performance, which we discuss here, with some reference to their educational implications.

## 1. Introduction

Multilingual education is a complex ecosystem arising from the interaction of linguistic, sociolinguistic, and educational variables [1,2]. This multidimensional nature has made it difficult to develop typologies of bi-/multilingual education that are simultaneously flexible and comprehensive [3,4,5,6,7,8]—see also [9] for review. Overall, these classifications have been deemed either too fixed to accommodate natural variability or too flexible and over-generating to yield typologies that correspond only to real-world examples. Among the many axes along which these models vary [10], socio-affective factors are some of the most prominent. In particular, much research has focused on characterizing variability among bi-/multilingual speakers in different contexts—including bilingual education—and has highlighted the role of speaker and family attitudes towards multilingualism [11].

Bilingual and multilingual education programs are not always implemented in social contexts where bi- or multilingualism is the norm—see chapters in [12]. While this is not, in and of itself, a barrier to the successful implementation of these programs, the distance between the linguistic reality of home and school environments might be perceived with insecurity by families and caregivers, who may feel that their inability to speak the language of instruction effectively prevents them from providing home assistance to their children. It is still unclear what the impact of these family attitudes might be on the linguistic development and language use of these pupils. However, some studies have shown that attitudes towards different languages and multilingualism more generally condition the use and time distribution that multilingual individuals make of their languages [13].

Before we continue, it is important to provide some short definitions of terms and concepts that appear recurrently throughout this article. First, by *attitudes* (towards language learning, multilingualism, and multilingualism-related practices), we mean the general outlook and qualitative evaluation a person makes of any aspect related to the subject matter. In the context of multilingualism and multilingual education, *attitudes* generally refer to the variably positive, negative, or somewhat neutral evaluation a person—typically a stakeholder, be it a parent or guardian, a family member, or the learner themselves—makes of the language being learned, the economic value of learning it, the social implications of acquiring and using this language, etc. *Perceptions*, which we also use at times, take on a similar meaning—with the semantic modulation of referring to a slightly more passive or receptive attitude. Our conception of *multilingualism* is multidimensional and not limited by factors such as age of onset (age of first exposure to a given language), relative grammatical or communicative competence between the different languages, literacy training, etc. Any person who, to some reasonable extent beyond anecdotal knowledge of a handful of words, speaks more than one language is considered a multilingual by our definition [14]. By convention, and since it is sometimes operationally important to make the distinction two-versus-many, we sometimes use *bilingualism* to refer to the knowledge and use of two languages, to the exclusion of *multilingualism*. Within what we call *multilingual practices*, that is, language habits that may only be considered in the context of multilingual communities, the most important is undoubtedly *language mixing*, *language switching*, or, our preferred term here, *code-switching*. By code-switching, we refer to systematic—code-switching has been shown to be rule-governed [15]—alternations between the languages of a multilingual, which may include changes across interlocutors, topics, sentences, or even words (intra-sentential code-switching). Other researchers have included code-switching under the more comprehensive terminological umbrella of *translanguaging* practices [16].

## 2. Literature Review

Kircher and colleagues [17] developed the new Attitudes Towards Childhood Multilingualism Questionnaire to gauge the evaluative dimensions of parental attitudes towards multilingualism in Quebec, Canada. Their work highlighted the fact that attitudes are not monolithic and can be factorized into several different dimensions. In particular, their results with 825 parents and caregivers of children aged 0–4 and raised in a multilingual home showed that attitudes clustered around at least three dimensions. Two of these dimensions are related to the consideration of language status and solidarity with respect to minority languages, which in most cases are heritage languages—home languages spoken in the context of a different, dominant societal language—in the context of Canada. Interestingly, these dimensions have interacted, in previous work, with a third: children’s cognitive development [18]. Parents who were speakers of heritage languages expressed more concerns about the negative consequences of raising their children multilingually (e.g., “inappropriate” language mixing and cognitive overload, among others). In a study by Kircher and colleagues, parents expressed mostly positive attitudes with respect to the relationship between multilingualism and cognitive development, stating their belief that multilingualism could make their children more flexible thinkers and potentially better learners.

Relatedly, research into multilingual language outcomes ([11,19]; among others) has taken up a quantification of code-switching practices—the alternating use of two languages, also known as *language mixing*—and attitudes towards multilingualism as compounded factors explaining much variability in multilingual linguistic competence and use. While this type of study is largely correlational and seeks to quantify and factorize the role of individual differences in defining and characterizing types of bilingualism, it is reasonable to make some inferences about the underlying causal relationships between attitudes and language outcomes. A strong candidate to explain why attitudes might affect competence and use in each language is the construct of *motivation*.

Research in second language acquisition (SLA) has dedicated much attention to attitude and motivation as key predictors of language learning success [20,21,22]. As with any construct, motivation presents challenges of operationalization that affect our quantification of its role in language learning. However, research in the past few decades strongly suggests that the (pre)disposition to learn foreign language users is modulated by several socio-affective and psycho-affective factors. The literature on motivation and SLA contains some mixed results (see, e.g., the meta-analysis in [23] for favorable evidence; cf. [24,25] for weaker correlations between achievement and motivation), which might be attributed to different operationalizations of the construct.

The attitudinal factors that modulate motivation have several origins. On the sociolinguistic side, considerations of language status have been shown to be important. In countries or regions with a multilingual population, majority languages (e.g., French in Brittany) and languages related to social and economic status (e.g., English) are usually regarded more favorably by learners, who show a higher motivation to study them [26,27]. The number of languages spoken also seems to have an impact on psycho-affective factors. While comparisons of bilinguals and multilinguals have not shown reliable differences in attitudes towards language learning itself [28], Dewaele and colleagues [29,30,31] have reported lower levels of communicative anxiety in multilinguals as compared with bilinguals, which is known to impact language learning—see, e.g., the *affective filter* in Krashen’s Monitor Model [32]. The reason behind this correlation might be the higher levels of communicative skills, socialization, and perceived competence reported for multilingual individuals [4,33].

The research reviewed above suggests that attitudes towards multilingualism are multidimensional and that their relationship to language outcomes is potentially stable. It is unclear, however, how the attitudes of the learners’ immediate environment (their family) can indirectly affect their success in language acquisition and how much this can, in turn, impact the overall academic performance of pupils enrolled in bilingual immersion programs. Recent developments in the public school agenda in Spain make the country a particularly interesting case study in which to analyze some of these questions. Of these developments, the most directly pertinent is the fast-growing spread of The British Council’s Bilingual Education Programme (BEP). The BEP started as a collaboration with the Spanish Ministry of Education and 10 Regional Governments and was originally based on the curriculum of the British Council School in Madrid. From its inception in 1996, the program has grown to cover around 40,000 students across the country through 90 primary schools and 58 secondary schools. While the program is now well established, there is still little information about the families of pupils attending these schools, especially regarding their views and practices on multilingualism. We believed that it was necessary to conduct a study that shed some light on the profiles of these families in Spain while simultaneously addressing some of the remaining questions about the relationship between family attitudes and academic performance in bilingual immersion programs. 

The present study focused on the attitudes towards multilingualism (as well as the language practices) of the families of 1001 children enrolled in the British Council’s BEP in public schools across Spain. Our three main objectives in this study were (1) to obtain a clearer picture of the family environment of children enrolled in bilingual immersion programs of this type, where the family and educational ecosystems are potentially very different; (2) to understand and characterize the multidimensionality of these families’ attitudes towards multilingualism; and (3) to assess potential correlations between these attitudinal factors and the overall academic performance of these pupils. To achieve this, we developed a tailored questionnaire gauging the linguistic practices of the family environment and their attitudes towards multilingualism and foreign language learning across several dimensions. Beyond these data that these answers provided for a better understanding of the family ecosystem, the relationship between family attitudes and the student’s academic performance was studied by examining potential correlations between the former and the pupils’ final grades in four different disciplines from the core BEP curriculum (Arts, Natural Science, Social Science, and Literacy/English).

## 3. Materials and Methods

### 3.1. Participants

One thousand and one families took part in this study by voluntarily completing the questionnaire that was distributed among the families of the schools of the BEP. In 816 cases, it was the mother of the child who completed the questionnaire. In 182 instances, it was the father, and in 3 cases, it was a legal guardian who was neither the father nor the mother of the child. Children did not complete the questionnaire. Overall, families of children attending 55 different schools following the BEP in 10 different Spanish autonomous communities took part in the study. Some of these schools were situated in bilingual regions (where, for at least some of the population, a vernacular language is spoken in addition to Spanish), but in no case was English a societal language for any of them. Five hundred and six of the children were girls, and the mean age of the sample was 10.76 years (SD = 1.06). The mean socio-economic status of the families measured on a 1–10 scale following the MacArthur Scale of Subjective Social Status [34] was 6.73 (SD = 1.30). All participants provided digital informed consent prior to starting the questionnaire, and the whole protocol of this study was approved by the Ethics Board of Nebrija University.

### 3.2. Materials

An online questionnaire was created, comprising a series of sociodemographic questions, followed by a self-perceived assessment of the English level of the child (a 1-to-10 proficiency scale to evaluate parental perceptions of children’s oral and written production and perception), a series of questions about the age and contexts of acquisition of English and an estimation of the number of minutes per day that the child would speak in English out of school with relatives or friends. The language background questionnaire also included questions regarding the preference of the family nucleus to use Spanish, English, or both at home and the level of satisfaction of the respondent with the child’s English level. The main questions of the language history, proficiency, and background questionnaire were adapted from the LHQ3 [35] and the Q-Bex [11]. An additional set of questions regarding the frequency of and attitudes towards language switching and mixing was included. To this end, respondents were asked to estimate the frequency at which language switching between the child and another interlocutor would occur at home and to indicate whether, according to their own view, language mixing in the same conversation was acceptable, undesirable, or something they did not have a formed opinion on. 

This questionnaire was then accompanied by a rating task in which respondents were presented with a list of 30 different statements that tapped into different categories of attitudes towards multilingualism, and they were asked to indicate using a 1-to-7 Likert-like scale their degree of agreement with the sentence (1 corresponding to “Totally disagree” and 7 to “Totally agree”). These statements were adapted from previous studies exploring attitudes toward multilingualism [17,36,37]. An English translation of the whole list of statements is provided in Appendix A. The whole questionnaire has been deposited as Supplementary Materials in an open repository that can be accessed via the following link: https://app.gorilla.sc/openmaterials/455332.

### 3.3. Procedure

All respondents were informed about the existence and nature of the study by their children’s corresponding school coordinators, who had had different informative meetings with the representatives of the British Council and Nebrija University prior to the start of the scientific actions. Participating families could access the questionnaire using any Internet-connected device following a link that was distributed. The questionnaire was prepared using Gorilla [38], and responses were collected using the same platform. Once these gathered data were curated and the final list of respondents determined, the research team contacted each school coordinator, asking for the grades on a 0-to-10 incremental scale of the children in the subjects that were being taught in English as part of the Bilingual Education Programme. Most of the children had Arts, Natural Sciences, Social Sciences, and Language/Literacy as the main school subjects in English, while some also reported having Physical Education, Maths, or Applied Knowledge. These grades were collected and averaged across school subjects for each child, constituting their mean score used as academic achievement in the analysis.

### 3.4. Data Coding and Analysis

Data from the first two parts of the questionnaire were processed and analyzed descriptively (without inferential statistics), as these were meant to provide a bird’s-eye view of the situation and demographics of these families in Spain. As for the attitudes towards multilingualism, the 30 statements were classified into 6 different categories prior to analyzing these data. These categories were (Socio)economic benefits of multilingualism, Cognitive benefits of multilingualism, Multilingualism as multiculturalism, Multilingualism and general learning, Multilingualism and social status, and Attitudes towards code-switching. Three independent raters classified each of the questions into possible categories. In the case of univocal agreement, a question would be clustered as part of the corresponding category. In case of partial agreement, the category provided by the majority of the raters was used. Inter-rater agreement was very high (70% corresponding to a Kappa = 0.755, z = 15.3, *p* < 0.001). Responses to statements referring to a positive attitude (e.g., “Learning English has cognitive advantages for the brain”) were kept as provided, and responses to statements referring to a negative attitude (e.g., “Mixing languages can affect school performance”) were transformed using an inversion of the response scale. This way, larger values always corresponded to more positive attitudes throughout the analysis. A reliability analysis was carried out to validate the 6 categories, and results showed a high goodness of consistency and reliability of these data once grouped by categories (Cronbach’s alpha = 0.85).

Across-category differences in ratings were analyzed statistically using an analysis of variance (ANOVA) and pairwise comparisons (more details in Section 4.3 below). The reason that these were not simply reported descriptively is that these statements on attitudes can be interpreted as motivations or incentives for these families to enroll or keep their children in a bilingual immersion program.

## 4. Results

### 4.1. Language History, Knowledge and Background

**Figure 1:**
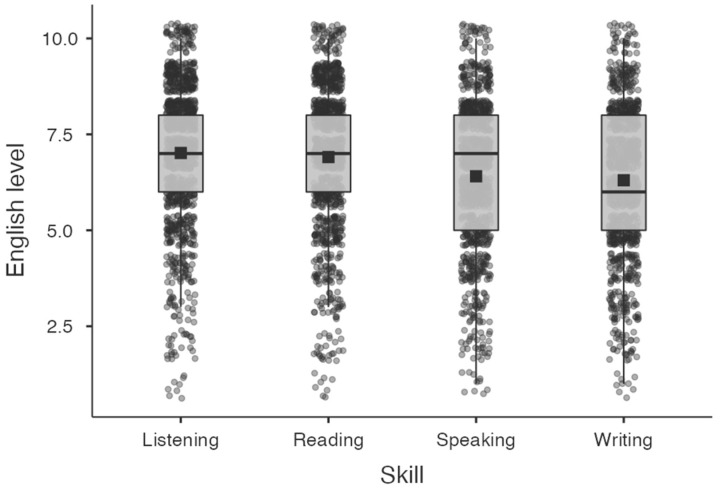
Box plots of the parent/guardian-rated English proficiency levels of students in the four basic language skills. The black square dots represent the mean, and the horizontal black lines represent the median. Listening corresponds to auditory comprehension. Reading corresponds to reading comprehension. Speaking corresponds to verbal production. Writing corresponds to written production.

The largest part of our sample had acquired English at the age of 3 or before (*n* = 879, representing 87.81% of the sample). When asked about the contexts in which English had been acquired, 212 families indicated that their children acquired English both at home and at school, 102 families reported that the context of English acquisition was exclusively at home, and 687 families indicated that English acquisition was done at school (see Table 1). When families were asked to rate their child’s English level, considering as a reference point that a person of the same age as their child who is a native English speaker would receive a score of 10 on a 1-to-10 scale, mean responses showed a medium-high proficiency (oral production: mean = 6.41, SD = 1.90; oral comprehension: mean = 7.01, SD = 1.92; written production: mean = 6.30, SD = 1.93; written comprehension: mean = 6.91, SD = 1.93). Figure 1 below graphically summarizes these ratings.

Responses to the number of minutes that the children spent every day using English to communicate with either the parents, other relatives, friends, and other adults were recorded using a 1-to-9 ordinal categorical scale (Nothing = 1, Less than 15 min = 2, 15 min = 3, 30 min = 4, 45 min = 5, 60 min = 6, 75 min = 7, 90 min = 8, More than 90 min = 9). The mean of responses showed very limited use of English outside school (mean = 1.62, SD = 0.87), roughly corresponding to less than 15 min of English use per day. When asked about what preference the members of the household showed for which language to use, most of the families responded that they never or almost never preferred to use English (n = 663, corresponding to 66.23% of the sample), or that they rarely preferred to use English (n = 193, 19.28%). Only 40 families (4%) reported that they often preferred to use English, and 12 families (1.20%) responded that they preferred to use English always or almost always. Ninety three families (9.29%) indicated that they had no preference at all. Finally, when asked about the level of satisfaction with their child’s ability to communicate in English following a 1-to-7 scale (1 being “Totally dissatisfied” and 7 being “Totally satisfied”), families reported a medium-high level of satisfaction (mean = 4.71, SD = 1.80). This information is summarized in Table 1 below.

### 4.2. Frequency and Opinions about Language Switching and Mixing

**Figure 2:**
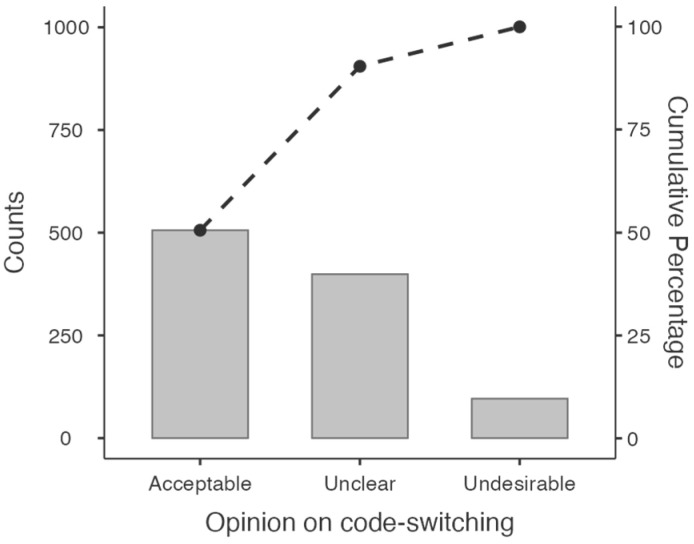
Pareto chart summarizing the respondents’ opinions on code-switching.

Families were asked about how often their children mixed languages either within a sentence or in the same conversation (across sentences) with family members, their friends, or other adults. They had to rate the frequency of language mixing following a 1-to-5 scale (1 being “Never” and 5 being “Always”), and mean responses indicated a very low prevalence of language-mixing habits (mean = 1.67, SD = 0.81). Families were also asked whether they felt that language mixing in the same conversation was acceptable, undesirable, or something they did not have a formed opinion on. Results showed that virtually half of the respondents (n = 505, corresponding to 50.45% of the sample) thought that language mixing was acceptable (see Figure 2). A very low number of families (n = 97, 9.69%) thought that language mixing was undesirable, and a large portion of the sample did not have any strong position in this regard (n = 399, 39.86%).

### 4.3. Attitudes towards Multilingualism

The mean polarity of the attitudes about the (Socio)economic benefits of multilingualism, as evidenced by the mean recorded responses to the six statements of this category (see Appendix A), was very positive (mean = 6.44, SD = 1.14). When considering the responses to the four statements classified under the category of Cognitive benefits of multilingualism, the mean value resulted in a generally positive view (mean = 6.00, SD = 1.49). The mean of the seven statements making up the category Multilingualism as multiculturalism also showed a generalized positive trend (mean = 5.99, SD = 1.58). The category Multilingualism and general learning included five statements, and the mean ratings of these items also showed a positive attitude (mean = 5.74, SD = 1.71). Responses to the six statements constituting the category Attitudes towards code-switching showed a mean value of 5.21 (SD = 1.97). Finally, responses to the two statements that together created the category labeled as Multilingualism and social status resulted in a mean rating of 5.14 (SD = 1.90). A statistical analysis of the differences between these mean scores per category was conducted using R [39] and *jamovi* [40], with the packages *afex* [41] and *PMCMR* [42]. The analysis of results per category showed that these data were not normally distributed (Shapiro-Wilk W > 0.74 and *p* < 0.001 across categories), and non-parametric Friedman repeated-measures ANOVA showed significant differences between the scores obtained in each category (c2(5) = 1682, *p* < 0.001). Durbin–Conover pairwise comparisons showed that the numerical differences observed in the mean ratings obtained across categories were endorsed by statistically significant differences (all *p*-values < 0.01), with the only exception of the scores in the Attitudes towards code-switching and Multilingualism and social status categories, which did not significantly differ from each other (*p* = 0.183).

### 4.4. Impact on Academic Achievement

**Figure 3:**
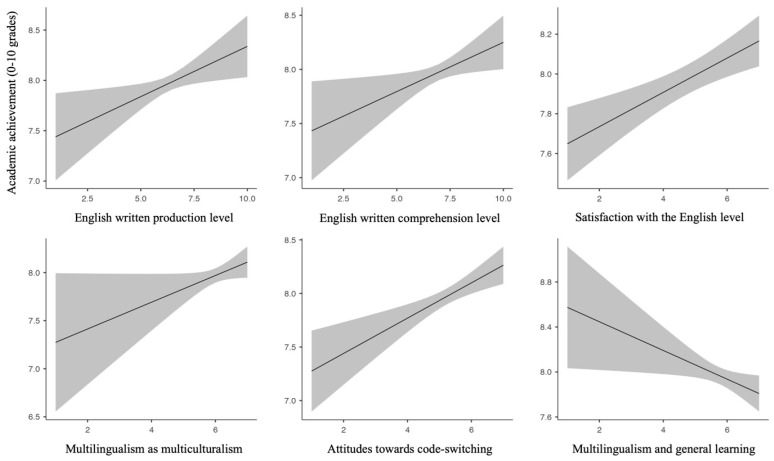
Plots of the estimated marginal means of the significant predictors for academic achievement. The grey areas correspond to 95% confidence intervals.

The mean grade across academic subjects and children was 7.97 (SD = 1.31) on a 0-to-10 scale. A stepwise regression analysis was performed in order to explore the extent to which the children’s English proficiency level perceived by the families and their degree of satisfaction with this level, as well as their attitudes towards multilingualism, could impact academic achievement. To this end, a first regression model was created, including the mean oral and written English production and comprehension level of the children (as perceived by the families and rated on a 1-to-10 scale) together with the families’ level of satisfaction with children’s ability to communicate in English (as rated following a 1-to-7 scale). A second model was constructed, including the mean responses to the statements classified per category (as evaluated using the 1-to-7 scale) as a second block. While the two models turned out significant (model 1: R = 0.35, adjusted R2 = 0.12, F(5, 995) = 28.0, *p* < 0.001; model 2: R = 0.42, adjusted R2 = 0.164, F(11, 989) = 18.9, *p* < 0.001), a model comparison confirmed that the 2-block model including the attitudes better accounted for the grades of the children (F(6, 989) = 9.96, *p* < 0.001). As presented in Table 2, the model coefficients showed that the academic achievement of the children could be significantly predicted by their written production (t = 2.44, *p* = 0.015) and comprehension levels (t = 2.34, *p* = 0.02), as well as by the level of satisfaction of their families with their English proficiency (t = 3.73, *p* < 0.001). Besides, more positive attitudes towards the (socio)economic benefits of multilingualism (t = 2.19, *p* = 0.029), the contribution of multilingualism to multiculturalism (t = 1.90, *p* = 0.057), and favorable attitudes towards code-switching (t = 3.68, *p* < 0.001) significantly predicted better academic achievement (see Figure 3). Curiously enough, the families’ beliefs that multilingualism could positively impact general learning were negatively related to the children’s achieved academic scores (t = −2.22, *p* = 0.027).

## 5. Discussion

The present study sought to paint a clearer picture of family attitudes and practices around multilingualism in the home environment of children enrolled in bilingual immersion programs in Spain and to gauge the role of these factors in the academic development of these students. To this end, we designed a questionnaire that focused on perceived competence and language practices, as well as on the attitudes towards multilingualism, and shared it among the family environment of students of the British Council’s Bilingual Education Programme (BEP) in schools across Spain. A total of 1001 parents and caregivers completed the survey. After a comprehensive description of both language practices and attitudes, we analyzed the relationship of these factors with the academic performance of the pupils, as measured by their average final grades in the subjects taught in English within the BEP. Our results showed that, while multilingual practices are not widespread in our sample, multilingualism is by and large perceived positively and considered to bring along benefits at different levels. As for the predictors of academic success, three factors related to subjective ratings of proficiency and three factors related to attitudes towards multilingualism and code-switching showed significant correlations with the student’s final grades. In what follows, we highlight some of the main aspects of these results. 

Perhaps the most notable aspect of those related to the families’ estimation of language outcomes and their report of language practices in the home is that these are, in most cases, not *multilingual* home environments as we typically understand them, or at least not with English playing a significant role—through active bilingualism with Spanish and vernacular languages is possible in a small number of cases from selected regions. Recall that only 23% of the families report *at least some* presence of English in the home. Unsurprisingly, language practices that are typical of bilingual communities, such as code-switching, are also reported to be rare: on a 1 to 5 scale (1 being “Never”), these children are reported to mix languages either across or within sentences at an average of 1.67. This is likely a result of the children’s competence and, especially, the largely monolingual status of these homes rather than any sort of coercion stemming from a negative view of code-switching: those who see this practice in a negative light barely make up 10% of the sample, and statements related to code-switching received an overall positive attitude, with an average of above 5 on a 1 to 7 scale.

To the extent that the statements on attitudes can be interpreted as motivations or incentives for these families to enroll or keep their children in a bilingual immersion program, it is important to look at how statistically significant differences in average ratings allow us to rank the statement categories. In this sense, it seems that parents and guardians see multilingualism, first and foremost, as a source of socio-economic and cognitive benefits for these children. They also believe that it is culturally enriching and that it might enhance learning more generally, and they are less emphatically positive (although still quite favorable) about its impact on social status and code-switching practices. Overall, these results are in line with recent studies on motivations for bilingual language practices in monolingual homes in Spain [43], where professional, social, cognitive, and educational incentives were ranked among the highest.

The correlations between language practices and attitudes and the student’s academic achievement provide some of the most interesting yet challenging results in our data. Among the six significant predictors, the three that are related to language competence are perhaps the easiest to explain. Considering that subjective ratings of language proficiency are a good proxy for general competence estimation [44], it is hardly surprising that the better a student performs in English, the better they perform overall in subjects taught in English. One key point to consider in this regard is that only the scores regarding the written comprehension and production levels had a predictive impact (and not the scores associated with the oral production and comprehension), probably as a result of the high reliance on the print for content transmission within the school. As for the attitudinal factors, they pose an interesting interpretive challenge. Most puzzling is the *negative* correlation between the families’ belief in the general learning benefits of multilingualism and their children’s academic performance. At the same time, there is no doubt that the statistical model points in this direction; we have no coherent explanation for why assuming better academic performance as a result of multilingualism may indeed correlate to worse academic performance. Causality is difficult to establish in such cases, however, and it might very well be the case that the families of already under-performing students are the ones with the most hope placed in multilingual education to improve their children’s academic achievements. In the absence of more data to illuminate this matter, however, we wish to refrain from speculating any further.

**Figure 4:**
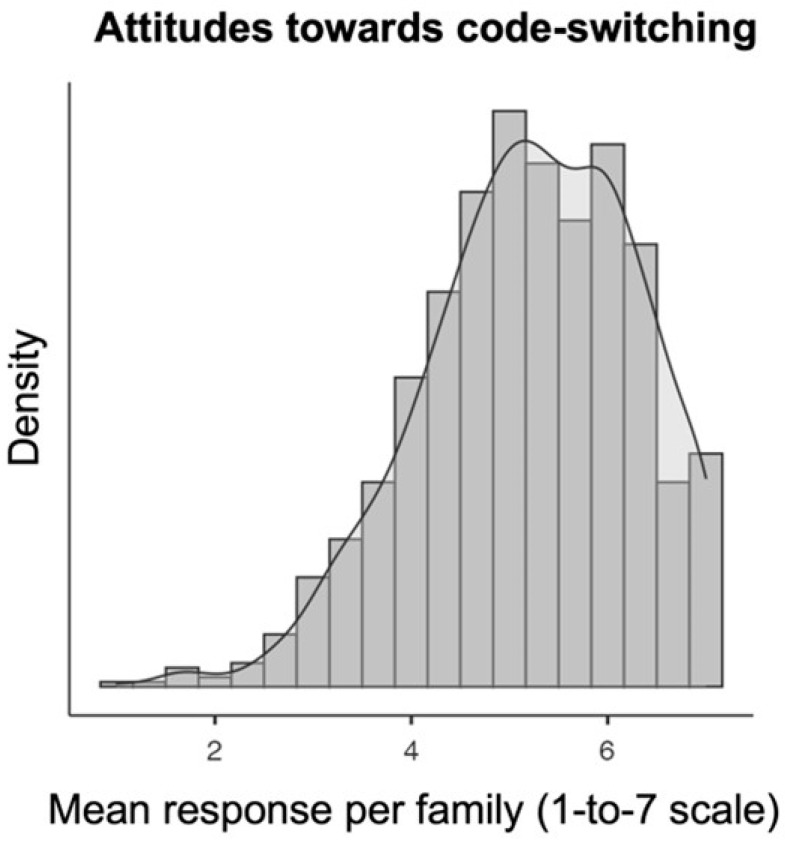
Density plot of the mean response per family (on a 1–7 scale) for the questions grouped under the ‘Attitudes towards multilingualism’ category. Higher bars signal more responses on a given point of the scale.

The two last factors that significantly predict academic performance are the statement categories of Multilingualism as Multiculturalism and Attitudes towards code-switching. In both cases, this correlation is a direct one: the more positive the attitudes in these two categories, the better the academic performance of the students. It is important, however, to consider more fine-grained estimations of these data. As we can see in Figure 4 below, responses to the latter category show a rather wide, positive-skewed distribution. This means that, although 50% of the families consider mixing acceptable and these statement categories average to at least 5 on a 1 to 7 scale, attitudes towards language switching and language mixing are heterogeneous. It is worth noting that the Attitudes towards code-switching category was the most influential variable in the regression model. This suggests that the more favorable parents and guardians are to language mixing, the better children’s academic performance is.

There are several practical implications that can be derived from this last point. The heterogeneity of attitudes towards code-switching, together with its role as the strongest predictor of academic achievement, suggests that there is still some work to be performed on the part of educators and language scientists to convey to the general public that code-switching and language-mixing practices are indeed a natural reality of multilingual communities. In fact, it is important to explain that not only has code-switching been proven *not* to have negative consequences, overall, but especially in the school environment [45,46], but it is indeed suspected to entail benefits for language production [47,48]. In this sense, translanguaging educational practices might be particularly beneficial for achieving a more active multilingual status [49,50,51] without any identifiable negative consequences for learning, language outcomes, or general cognition. 

As for the limitations of this study, our questions on language practices, which constitute an important part of the questionnaire, are naturally self-reported, and as such, they provide information on what families *think they do* rather than what *they actually do*—and there might be discrepancies between the two. This is a good example of the kind of data granularity that is traded off in large-scale studies and highlights the importance of smaller-scale, more focused investigations to complement our understanding of these phenomena. In terms of statement polarity, one might wonder whether a better balance of positively- and negatively-formulated statements in certain categories (more notably, 1 and 5) could yield different results. While we do not believe that modifications in polarity should significantly change the overall results of the study, we acknowledge that better balance would be desirable in future work to avoid or at least partially counteract acquiescence biasing.

This study, the first of this scale in Spain, has provided a clearer picture of the family environment of children enrolled in the British Council’s Bilingual Education Programme and has contributed to shedding light on the type of attitudinal factors that are most closely related to the student’s academic performance in this type of programs. Future research should investigate further to establish the nature and directionality of any potential causal links and derive implications for the classroom and the overall dynamics of these school ecosystems in order to maximize academic performance and, more generally, improve the students’ experience in bilingual immersion programs. In this sense, it might be interesting to investigate the relationship between these attitudes and academic performance overall (not just in English-medium subjects) in dual-language programs with a better balance between languages to remove confounds related to sheer comprehension of content and obtain a more nuanced account of how multilingual competence may impact school performance.

## Figures and Tables

**Table 1:** Summary of questions relating to language background and practices.

Question	Response	Amount/Percentage	Mean	SD
First exposure to English	Before/at age 3	879 (88%)	-	-
	After age 3	122 (22%)	-	-
Context of English exposure	Only at home	102 (10%)	-	-
	Only at school	687 (69%)	-	-
	Both home and school	212 (21%)	-	-
Use of English outside school	1 = Nothing; 2 = < 15 min; 3 = 15 min; 4 = 30 min; 5 = 45 min; 6 = 60 min; 7 = 75 min; 8 = 90 min; 9 = > 90 min.	-	1.62	0.87
Preferred language at home	Never/almost never English	663 (66%)	-	-
	Rarely English	193 (19%)	-	-
	Often English	40 (4%)	-	-
	Always English	12 (1%)	-	-
	No preference	93 (9%)	-	-
Satisfaction with child’s English level	1 = Totally dissatisfied … 7 = Totally satisfied	-	4.71	1.80

**Table 2:** Regression model coefficients.

Predictor	Estimate	SE	*t*	*p*
* Intercept *	4.19	0.36	11.62	<0.001
Oral production	0.03	0.04	0.68	0.496
Oral comprehension	−0.03	0.04	−0.66	0.508
Written production	0.10	0.04	2.44	0.015
Written comprehension	0.09	0.04	2.34	0.020
Satisfaction w/English level	0.09	0.02	3.73	<0.001
Category 1: Socio-economic benefits	0.16	0.07	2.19	0.029
Category 2: Cognitive benefits	0.05	0.06	0.89	0.373
Category 3: Multiculturalism	0.14	0.07	1.90	0.057
Category 4: General learning	−0.13	0.06	−2.22	0.027
Category 5: Attitudes towards code-switching	0.16	0.04	3.68	<0.001
Category 6: Social status	−0.04	0.03	−1.11	0.270

Note. Category 1: (Socio)economic benefits of multilingualism; Category 2: Cognitive benefits of multilingualism; Category 3: Multilingualism as multiculturalism; Category 4: Multilingualism and general learning; Category 5: Attitudes towards code-switching; Category 6: Multilingualism and social status.

## Data Availability

The data presented in this study are available on request from the corresponding author. Data and analysis code are available upon request.

## Supplementary Materials

The full questionnaire can be accessed at https://app.gorilla.sc/openmaterials/455332.

## Appendix A

Statements on attitudes towards multilingualism were included in the questionnaire. Statements are presented here sorted by category following the pre-analysis classification, although they were presented to participants unclassified and in a randomized order.

1. *(Socio)economic benefits of multilingualism*
  a. In our society it is important to speak several languages.
  b. Speaking English opens the door to a higher quality education.
  c. Speaking English makes it easier to navigate the Internet and social networks.
  d. Speaking English improves the chances of finding a job.
  e. Speaking more than one language can help in today’s competitive job market.
  f. A good level of English provides more opportunities for advancement in the workplace.
2. *Cognitive benefits of multilingualism*
  a. Learning English has cognitive advantages for the brain.
  b. Growing up speaking several languages can be confusing for a young child.
  c. Speaking more than one language will help my child think more flexibly.
  d. Speaking more than one language will improve my child’s reasoning ability.
3. *Multilingualism as multiculturalism*
  a. Learning English is a form of multiculturalism.
  b. Communicating well in English allows you to meet more people.
  c. English is useful when traveling.
  d. Multilingualism hinders communication and creates barriers to understanding.
  e. The growing presence of English in social networks, the media and other areas is detrimental to the country’s cultural identity.
  f. Mixing languages denotes a multicultural identity.
  g. A multilingual society is a culturally richer society.
4. *Multilingualism and general learning*
  a. Over time, speaking more than one language will help my child get better grades in school.
  b. Speaking more than one language helps in learning other languages.
  c. Speaking more than one language will make my child a more capable and efficient learner.
  d. Teaching in English lowers the level and affects the content of the subjects.
  e. Having knowledge of English helps when handling electronic devices.
5. *Attitudes towards code-switching*
  a. Mixing languages can affect school performance.
  b. Languages should be learned one at a time, not all at once.
  c. It bothers me when people mix and switch between languages I don’t know when I am present.
  d. Mixing languages shows arrogance on that person’s part.
  e. Mixing languages shows that the person is not really proficient in one or any of the languages.
  f. Mixing languages is a way of showing solidarity with a culture.
6. *Multilingualism and social status*
  a. Bilingual education programs promote elitism and increase inequality
  b. Multilingual people generally have a high social status.

## References

[1] Cenoz J., Gorter D. (2023). Multilingualism at School and Multilingual Education.

[2] Rothman J., González Alonso J., Puig-Mayenco E. (2019). Third Language Acquisition and Linguistic Transfer.

[3] Baetens Beardsmore H. (1993). An overview of European models of bilingual education. Lang. Cult. Curric..

[4] Baker C. (2006). Foundations of Bilingualism and Bilingual Education.

[5] Cummins J., Dewaele J.-M., Housen A., Wei L. (2003). Bilingual education: Basic principles. Bilingualism: Beyond Basic Principles.

[6] Hornberger N.H., García O. (1991). Extending enrichment bilingual education: Revisiting typologies and redirecting policy. Bilingual Education.

[7] Hornberger N.H., Creese A., Martin P., Hornberger N.H. (2007). Continua of biliteracy. Encyclopedia of Language and Education. Vol. 9: Ecology of Language.

[8] Ytsma J. (2001). Towards a typology of trilingual primary education. Int. J. Biling. Educ. Biling..

[9] Edwards J., Auer P., Wei L. (2007). Societal multilingualism: Reality, recognition and response. Handbook of Multilingualism and Multilingual Communication.

[10] Cenoz J. (2009). Towards Multilingual Education.

[11] De Cat C., Kašćelan D., Prevost P., Serratrice L., Tuller L., Unsworth S. (2021). Quantifying Bilingual EXperience (Q-BEx): Questionnaire Manual.

[12] García O. (2008). Bilingual Education in the 21st Century: A Global Perspective.

[13] Jongbloed-Faber L., Van der Velde H., van der Meer C. (2016). Language use of Frisian bilingual teenagers on social media. Treb. Socioling..

[14] Rothman J., Bayram F., DeLuca V., Di Pisa G., Dunabeitia J.A., Gharibi K., Hao J., Kolb N., Kubota M., Kupisch T. (2022). Monolingual comparative normativity in bilingualism research is out of “control”: Arguments and alternatives. Appl. Psycholinguist..

[15] Parafita Couto M., Bellamy K., Ameka F.K., Cabrelli J., Chaouch-Orozco A., González Alonso J., Pereira Soares S.M., Puig-Mayenco E., Rothman J. (2023). Theoretical Linguistic Approaches to Multilingual Code-Switching. The Cambridge Handbook of Third Language Acquisition and Processing.

[16] Wei L. (2018). Translanguaging as a practical theory of language. Appl. Linguist..

[17] Kircher R., Quirk E., Brouillard M., Ahooja A., Ballinger S., Polka L., Byers-Heinlein K. (2022). Quebec-based Parents’ Attitudes Towards Childhood Multilingualism: Evaluative Dimensions and Potential Predictors. J. Lang. Soc. Psychol..

[18] Ballinger S., Brouillard M., Ahooja A., Kircher R., Polka L., Byers-Heinlein K. (2022). Intersections of official and family language policy in Quebec. J. Multiling. Multicult. Dev..

[19] Anderson J.A., Mak L., Keyvani Chahi A., Bialystok E. (2018). The language and social background questionnaire: Assessing degree of bilingualism in a diverse population. Behav. Res. Methods.

[20] Dörnyei Z., Ushioda E. (2011). Teaching and Researching Motivation.

[21] Ushioda E. (2016). Language learning motivation through a small lens: A research agenda. Lang. Teach..

[22] Ushioda E., Dörnyei Z., Gass S., Mackey A. (2012). Motivation. The Routledge Handbook of Second Language Acquisition.

[23] Masgoret A.-M., Gardner R.C. (2003). Attitudes, motivation, and second language learning: A meta-analysis of studies conducted by Gardner and associates. Lang. Learn..

[24] Lasagabaster D. (1998). The Threshold Hypothesis applied to three languages in contact at school. Int. J. Biling. Educ. Biling..

[25] Vandergrift L. (2005). Relationships among motivation orientations, metacognitive awareness and proficiency in L2 listening. Appl. Linguist..

[26] Bernaus M., Masgoret A.-M., Gardner R.C., Reyes E. (2004). Motivation and attitudes towards learning languages in multicultural classrooms. Int. J. Multiling..

[27] Mettewie L., Janssens R., Lasagabaster D., Huguet À. (2007). Language use and language attitudes in Brussels. Multilingualism in European Bilingual Contexts: Language Use and Attitudes.

[28] Brohy C. (2001). Generic and/or specific advantages of bilingualism in a dynamic plurilingual situation: The case of French as official L3 in the School of Samedan (Switzerland). Int. J. Biling. Educ. Biling..

[29] Dewaele J.-M. (2002). Psychological and sociodemographic correlates of communicative anxiety in L2 and L3 production. Int. J. Biling..

[30] Dewaele J.-M. (2007). The effect of multilingualism, sociobiographical, and situational factors on communicative anxiety and foreign language anxiety of mature language learners. Int. J. Biling..

[31] Dewaele J.-M., Petrides V.K., Furnham A. (2008). Effects of trait emotional intelligence and sociobiographical variables on communicative anxiety and foreign language anxiety among adult multilinguals: A review and empirical investigation. Lang. Learn..

[32] Krashen S., Terrell T. (1988). The Natural Approach: Language Acquisition in the Classroom.

[33] Wolff H.E. (2000). Pre-school child multilingualism and its educational implications in the African context. PRAESA—Occas. Pap..

[34] Adler N.E., Epel E.S., Castellazzo G., Ickovics J.R. (2000). Relationship of subjective and objective social status with psychological and physiological functioning: Preliminary data in healthy, White women. Health Psychol..

[35] Li P., Zhang F., Yu A., Zhao X. (2020). Language History Questionnaire (LHQ3): An enhanced tool for assessing multilingual experience. Biling. Lang. Cogn..

[36] Dewaele J.-M., Wei L. (2014). Attitudes towards code-switching among adult mono- and multilingual language users. J. Multiling. Multicult. Dev..

[37] Purschke C. (2020). Attitudes Toward Multilingualism in Luxembourg. A Comparative Analysis of Online News Comments and Crowdsourced Questionnaire Data. Front. Artif. Intell..

[38] Anwyl-Irvine A.L., Massonnié J., Flitton A., Kirkham N., Evershed J.K. (2019). Gorilla in our midst: An online behavioral experiment builder. Behav. Res. Methods.

[39] R Core Team (2021). R: A Language and Environment for Statistical Computing, Version 4.0; [Computer Software]. https://cran.r-project.org.

[40] The Jamovi Project (2021). Jamovi, Version 2.2; [Computer Software]. https://www.jamovi.org.

[41] Singmann H. (2018). afex: Analysis of Factorial Experiments. [R Package]. https://cran.r-project.org/package=afex.

[42] Pohlert T. (2018). PMCMR: Calculate Pairwise Multiple Comparisons of Mean Rank Sums. [R Package]. https://cran.r-project.org/package=PMCMR.

[43] Nogueroles López M., Pérez Serrano M., Duñabeitia J.A. (2022). Hablar con tu(s) hijo/a(s) en lengua extranjera: Motivaciones de las familias. Estud. Linguist. Ingl. Apl. (ELIA).

[44] Marian V., Blumenfeld H.K., Kaushanskaya M. (2007). The Language Experience and Proficiency Questionnaire (LEAP-Q): Assessing language profiles in bilinguals and multilinguals. J. Speech Lang. Hear. Res..

[45] Antón E., Duñabeitia J.A. (2021). *¡Hola! Nice to Meet You:* Language Mixing and Biographical Information Processing. Brain Sci..

[46] Antón E., Thierry G., Duñabeitia J.A. (2015). Mixing Languages during Learning? Testing the One Subject—One Language Rule. PLoS ONE.

[47] de Bruin A., Samuel A.G., Duñabeitia J.A. (2018). Voluntary language switching: When and why do bilinguals switch between their languages?. J. Mem. Lang..

[48] Gollan T.H., Ferreira V.S. (2009). Should I stay or should I switch? A cost-benefit analysis of voluntary language switching in young and aging bilinguals. J. Exp. Psychol. Learn. Mem. Cogn..

[49] Canagarajah S. (2011). Codemeshing in Academic Writing: Identifying Teachable Strategies of Translanguaging. Mod. Lang. J..

[50] Cenoz J., Gorter D. (2017). Minority languages and sustainable translanguaging: Threat or opportunity?. J. Multiling. Multicult. Dev..

[51] García O., Wei L., García O., Wei L. (2014). Language, bilingualism and education. Translanguaging: Language, Bilingualism and Education.

